# A comprehensive review on recent advances in the use of ethnomedicinal plants and their metabolites in snake bite treatment

**DOI:** 10.3389/fphar.2025.1548929

**Published:** 2025-03-12

**Authors:** Ashish Kumar, Rameshwari A. Banjara, Roman Kumar Aneshwari, Junaid Khan, Paulo Sergio Bernarde

**Affiliations:** ^1^ Department of Biotechnology, Guru Ghasidas Vishwavidyalaya (A Central University), Bilaspur, Chhattisgarh, India; ^2^ Department of Chemistry, Rajeev Gandhi Government Postgraduate College, Ambikapur, Chhattisgarh, India; ^3^ School of Pharmacy, MATS University, Raipur, Chhattisgarh, India; ^4^ Department of Pharmacy, Sant Gahira Guru Vishwavidyalaya, Sarguja Ambikapur, Chhattisgarh, India; ^5^ Laboratório de Herpetologia, Centro Multidisciplinar, Campus Floresta, Universidade Federal do Acre, Cruzeiro do Sul, Acre, Brazil

**Keywords:** snakebite, snake venom, ethnomedicine, medicinal plants, phytoconstituents

## Abstract

Snakebites are a severe medical and social issue, particularly in tropical and subtropical countries with minimal medical facilities, where the most dangerous snakes are found. Worldwide, most rural areas use medicinal plants alone or in combination as antidotes for snakebite treatment. Local knowledge of medicinal plants for snakebite treatment plays a more critical role in primary healthcare services in rural areas. As a result of this review, it is revealed that 39% of herbs, 38% of shrubs, 18% of trees, 2% of climbers, 2% of bulbs, and 1% of ferns have snake antivenom potential, which is indicative of the presence of numerous phytochemicals such as alkaloids, coumarins, curcuminoids, flavonoids, steroids, triterpenoids, and cinnamic acid in particular plants. According to the availability of information, the data focus on the plants, their families, and their parts from various literature sources. In the future, the valuable plants reported here and their phytoconstituents may be potential sources for developing effective natural drugs for snake bite treatments. Therefore, this review is a comprehensive study of the snake antivenom potential of various medicinal plants and their bioactive compounds.

## 1 Introduction

Snake bites are a public health hazard; according to a World Health Organization (WHO) estimate (World Health Organization, 2021), up to 2.5 million instances of envenomation are caused yearly by snakebites. In many developing countries, snakebite is a severe issue, i.e., India is the country where the most number of snake bites occurs; the number of bites reaches about one million per year with about 600,000 envenomations and 58,000 deaths per year (Suraweera et al., 2020). The most commonly used therapeutic agent for snakebite treatment is the antiserum (antivenom), which is conventionally prepared by injecting a non-lethal quantity of snake venom into mammals such as rabbits and horses to raise immunoglobins against snake venom, followed by separation of serum containing the immunoglobins from the mammal’s blood (Makhija and Khamar, 2010) In addition to several studies, plant substances have been recognized as a good source of snake venom neutralization and claimed to be an antidote for snakebite treatment (Houghton and Skari, 1994; Abubakar et al., 2000). Numerous *in vitro* and *in vivo* studies reported in the literature that bioactive metabolites extracted and produced from medicinal plants exhibit antivenom properties (Gutiérrez et al., 2021). Worldwide, especially in rural areas, different parts of plant extracts have traditionally been used for snakebite treatment alone or in combination. The ethnomedicinal studies reported that around 116 plants, including several trees, herbs, shrubs, climbers, bulbs, and ferns, have snake antivenom potential. Thus, in this review article, we have enumerated some ethnomedicinal plants possessing snake antivenom potential and elucidated their phytoconstituents and mechanisms of action for snakebite treatment.

## 2 Venomous snake

Among 3,848 snakes found worldwide, around 750 are venomous (Uetz et al., 2020). Particularly in India four species *Bungarus caeruleus* (common krait), *Naja naja* (spectacled cobra), *Echis carinatus* (saw-scaled viper), and *Daboia russelii* (Russell’s viper) of more than 60 venomous species are the leading cause of bites and resulting morbidity (Sulabh and Shivahre, 2018; Senji Laxme et al., 2019).

### 2.1 Complications originated in response to snake venom

Snake venom is a complex fusion of phospholipase A2 (PLA2s), myotoxins, hemorrhagic metalloproteinases, other proteolytic enzymes, coagulants, cardiotoxins, cytotoxins and neurotoxins, enzymes, and toxic proteins (Soares et al., 2004). Snake envenomation can have various clinical consequences, such as systemic and local pathology. Serious problems can arise from snakebite complications; in the cases of viper bites, the majority of patients (17/24) were diagnosed with intracranial hemorrhage and ischemic stroke, and in some instances, there may be cerebral complications also (Huang et al., 2022). Similarly, *Echis carinatus* envenomation results in necrosis, hemorrhage, blistering, and swellings due to the occurrence of both caring and other metalloproteases in the snake venom (Jayawardana et al., 2018), whereas *Naja nigricollis* envenoming results in local necrosis, complement depletion, hemorrhage, and respiratory arrest or paralysis (Rivel et al., 2016). *Naja nigricollis* venom consists of cardiotoxin and phospholipase A2s (an anticoagulant enzyme that binds to coagulation factor Xa and inhibits the prothrombinase complex).

In some cases, anterior uveitis and corneal ulceration may result from poisoning. Envenomation of *Bothrops venom* in humans causes damage to local tissues, edema, hemorrhage, myonecrosis, and proteolysis. Due to multiple snake envenomation, muscle necrosis is a significant local consequence, often leading to irreversible tissue loss (Rucavado et al., 2005; Bittenbinder et al., 2024). In addition, as a result of vessel degeneration and hemorrhagic metalloproteases-induced ischemia, myonecrosis can evolve as an indirect action or a direct effect of myotoxic homologous PLA2s on muscle cell plasma membranes (Gutierrez et al., 2009). It is also reported that the venom’s composition determines the type of problems, which differs by geographic location (Narvencar et al., 2022).

### 2.2 Limitations of antisnake venom therapy

The only reliable treatment available against snake venom poisoning is antivenom immunotherapy. However, several side effects, such as pyrogen reaction, serum sickness, and anaphylactic shock, are associated with this antivenom immunotherapy. Such symptoms can mainly arise due to the action of highly concentrated non-immunoglobulin proteins in commercially existing hyperimmune antivenoms (Makhija and Khamar, 2010). However, intravenous antivenom administration prepared from the Immunoglobulin G(IgG) of sheep or horses has been used as the most effective remedy for systemic envenoming of victims. The clinical limitation of snake poisoning treatment with antivenom is the lag time in response to the effect of envenomation, which develops rapidly after a bite. Consequences of such envenomation include extreme pain, necrosis, edema, and localized hemorrhage; this leads to deformity and lifelong scarring (Makhija and Khamar, 2010). Specially antivenoms manufactured in India are only against the four main venomous snake species (*D. russelii*, *B. caeruleus*, *Naja naja, and E. carinatus*) and are not able to neutralize the venom of some species (Senji Laxme et al., 2019). In addition to a better quality antivenom, there is also a need for better training of doctors in snakebite management, thus reducing mortality (Simpson, 2008).

## 3 Ethnobotanical for the treatment of snakebite

As ethnobotanicals are inexpensive and easily accessible, they are widely used by indigenous people for snakebite treatment (Gomes et al., 2010). In rural areas, herbal medicine without antivenom therapy against envenomation is generally accepted among ethnic groups. Plant extracts provide an extraordinarily rich source of pharmacologically active bioactive metabolites and have numerous pharmacological properties. Interaction of such metabolites with toxins or enzymes neutralizes and inhibits their activities (Adrião Asenate et al., 2022). So, plant-based remedies are significant for snakebite treatment and can find suitable alternatives to antivenom immune therapy. Venomous snakes are found in almost all parts of the world; many plant materials are used as traditional medicine for snakebite treatment (Hussain and Kingsley, 2024). Generally, an aqueous extract of methanol and ethanol from plants is prepared for medical application. Plant extract of medicinal value is administered via different routes, for example, the topical solution applied to the snakebitten area, ingestion of the decoctions plant extract, chewing leaves, and barks, etc. snakewise uses of traditional herbs, their plant extract, and ad method of administration are reviewed by David et al., 2022.

Since ancient times, the plant roots of *Ophiorrhiza mungo*, *Gymnema sylvestre*, *Peristrophe bicalyculata*, *Cucumis colosynthis*, *Gloriosa Superba*, *Alangiumsi salvifolium*, *Enicostemma axillare*, and *Aristolochia indica* leaves have been used in ayurvedic medicine. According to the Ayurveda system, specific plant species may be used against specific snakebites, e.g., In the treatment of krait bite, *Abrus precatorius* root extracts have been used; Azadirachta indica leaf paste is used against viper bites in addition to rock salt (Shukla et al., 2010). Casearia sylvestris bark and leaves have been used as an ideal ayurvedic medicine for snakebite treatment in the Columbia and Indian subcontinents for a long time.

Knowles (1921), published the first plant-based antidote. He screened some phytoconstituents from plants used by traditional healers. Still, unfortunately, he failed to define their efficacy against snake envenomation due to the sublethal or non-lethal dosage of venom. Further, 314 plants and 184 combinations were screened for lethality, ignoring the systemic changes caused by snake venom and advocating the efficacies of herbal antidotes (Mhaskar and Caius, 1931). It has been investigated that *N. naja* venom is neutralized, and the hemorrhage effect due to *Vipera russelli* and *Trimeresurus flavoviridis* venoms are further reduced by *Aristolochia species* ether soluble plant substances (Vishwanath et al., 1987). In mice, rhizomes of *Curcuma* species inactivated the postsynaptic neurotoxin of Thai kobra (*N. naja* siamensis) (Ratanabanangkoon et al., 1993). *S. magnificum bark*, *Mucuna pruriens var*. utilis, *Strophanthus gratus*, and *S. hispidus* leaves aqueous extract increases the clotting time with a standardized dose of *E. carinatus* snake venom (Houghton and Skari, 1994). Compared to the other group of mice treated with *E. carinatus* and *N. nigricollis* snake venom, the survival period of male albino mice was increased by the application of Guiera senegalensis leaf extracts (Abubakar et al., 2000; Otero et al., 2000 have reported that stem bark extract of *Tabebuia rosea, Brownea rosademonte, Trichomanes elegans whole plants, Heliconia curtispatha* rhizomes, and *Bixa orellana, Gozalagunia panamensis, Struthanthus orbicularis, Philodendron tripartitum* branches and leaves and the ripe fruit of Citrus lemon, leaves, stems and branches of Ficus nymphaeipholia inhibited the hemorrhage (Otero et al., 2000).

In addition (Otero et al., 2000), have reported that plant extracts of *Aristolochia grandiflora, Columnea kalbereyeriana, Sida acuta, Selaginella* sp rhizomes of *Renealmia alpinia*, the stem of *Strychnos xynguensisleaves*, branches of *Hyptis capitata, Ipomoea cairica, Ocimum micranthum, Piper pulchrum, Neurolaena lobata Castilla elastica, Siparuna thecaphora, Allamanda cathartica, Capsicum frutescens* macerated fruits, unripe fruit of *Crescencia cujete*, branches and leaves of *Passiflora quadrangularis* and *Piper arboretum* partially inhibited hemorrhage caused by snake venom. Aguiyi et al., 2001 have recorded that *M. pruriens* var. utilis seeds aqueous extract inhibits venom-induced mycotoxin, cytotoxic coagulation in experimental animals caused by *E. carinatus* snake.

Alcoholic and aqueous extracts of dried roots of *Mimosa pudica* inhibit myotoxicity and lethal effects induced by toxic enzymes of *Naja kaouthia* venom (Mahanta and Mukherjee, 2001). *M. pudica* also possesses anti-hyaluronidase activity against *N. naja*, *V. russelii*, and *E. carinatus* venom (Girish et al., 2004). Furthermore, the butanol extract of *M. pudica* and *Eclipta prostrata* showed therapeutic significance, as it partially inhibits phospholipase A2 and hemorrhage induced by the proteolytic activity of Malayan pit viper venom (Pithayanukul et al., 2004). Oral administration of garlic was used as a prophylactic alternative for cobra venom-induced histochemical patterns and histological changes of the hepatic and gastric tissues in the rats (Rhamy and Hemmaid, 2001). A water-methanol extract from stems of Parkia biglobosa can neutralize *N. nigricollis* and *E. ocellatus* snake venom in some experimental models (Asuzu and Harvey, 2003). Partial inhibitions of *Bothrops* and *Crotalus durissus terrificus* venoms' phospholipase function were shown by crude aqueous extract from *Mandevilla velutina* (Biondo et al., 2003). The *Marsypianthes change* dry extract from the Brazilian plant inhibits fibrinogen clotting caused by the Brazilian snake venom, suggesting its role in affecting thrombin-like enzymes (Castro et al., 2003). *Casearia sylvestris* aqueous extract has shown anti-phospholipase A2 (PLA2), myotoxic, and hemorrhagic activities induced by crude snake venoms and toxins (Raslan et al., 2002). In addition, *Casearia mariquitensis* neutralizes haematological and systemic alterations caused by Bothrops neuwiedi pauloensis venom (Izidoro et al., 2003). *Mandevilla illustris* inhibited *C. durissus* terrificus snake venom phospholipase activity and increased the survival time of patients (Biondo et al., 2004). *Struthanthus orbicularis*, *B. orellana, Ficus nymphaeifolia, Gonzalagunia panamensis* branches and leaves, *T. rosea, B. rosademonte* stem and barks, *T. elegans* and *Pleopeltis percussa* whole plant, *H. curtispatha*, *R. alpinia*, and *Dracontium croatii* rhizomes and the ripened fruits of *Citrus lemon* inhibit the defibrination, edema, and coagulation caused by *Bothrops asper* venom (Nunez et al., 2004).

The ethanol root extract of *Acalypha indica L.* has a potent snake venom-neutralizing ability (Shirwaikar et al., 2004). Aqueous extract of *Tabernae montana* catheriensis prevented the lethal effect induced by *C. durissus terrificus* snake venom (de Almeida et al., 2004). It partially inhibited the myotoxic effects of *B. jararacussu* venom containing two myotoxins, bothropstoxin-1(BthTX1) and bothropstoxin-2(BthTX2), with low PLA2 activity (Veronese et al., 2005). The methanol extract of *Annona senegalensis* Pers root bark caused a decrease in the *N. nigricollis* venom-induced hyperthermia in rats (Adzu et al., 2005). *In vitro, Musa paradisiaca* L. successfully inhibited viper venom actions e (Borges et al., 2005). *Pentaclethra macroloba* exhibited complete neutralization of hemorrhagic and nucleolytic activities caused by several snake venoms and partial inhibition of myotoxic, phospholipase, lethal, and edema activities. It neutralized *Bothrops jararacussu* metalloprotease-induced hemorrhage in the *in-vivo* model (daSilva et al., 2005). Aqueous extract of *Croton urucurana* containing proanthocyanidins reduced the hemorrhagic activity of *B. jararaca* venom (Esmeraldino et al., 2005). Aqueous extract of fresh roots, leaves, and stems of *Mikania glomerata* efficiently inhibited different pharmacological, toxic, and enzymatic effects caused by venoms from *Bothrops* and *Crotalus* snakes (Maiorano et al., 2005). *Cordia Verbenaceae* neutralized paw edema induced by *B. jararacussu* snake venom (Ticli et al., 2005). Aqueous extract of aerial parts of *Bauhinia fortification* is a source of natural inhibitors of serine proteases participating in blood clotting, disturbances induced by *Bothrops* and *Crotalus* crude venoms (Oliviera et al., 2005). The methanol bulb extract of *Crinum jagus* significantly prevented mice from hemorrhage, myonecrosis, and death induced by a lethal dose of *E. ocellatus*, *Bitis arietans*, and *N. nigricollis* venoms (Ode and Asuzu, 2006).

Tamarind seed extract inhibited the PLA2, hyaluronidase, protease, amino acid oxidase, and 5-nucleotidase enzyme (major hydrolytic enzymes) activities of *Vipera russelii* venom in a dose-dependent manner. Additionally, the extract inhibited indirect hemolysis by venom and the degradation of the human fibrinogen B-chain. The extract showed a moderate effect on clotting time. Edema, myotoxic, hemorrhage effects, and lethality caused by venom were significantly inhibited when different doses of the extract were preincubated with venom before assays. On the other hand, animals that received extract 10 min after the injection of venom recovered from toxicity caused by the venom (Ushanandini et al., 2006). Dichloromethane extract from leaves of *Artemisia campestris* inhibited the venom-induced actions of viper *Macrovipera lebetina* (Memmi et al., 2007). Ethanol extract of *Galactia glaucescens* inhibited the neuromuscular paralysis caused by *C. durissus terrificus* venom (Dal Belo et al., 2008). Edema, myonecrosis, and hemorrhage coagulation caused by Indian *E. carinatus* (saw-scaled viper) venom were inhibited by the methanol seed extract of *Vitis vinifera* L. (Mahadeswaraswamy et al., 2008). The aqueous extract of *Schizolobium parahyba* displayed potent antivenom ability (Mendes et al., 2008; Vale et al., 2008). The active fractions of *Aristolochia indica*, *G. superba*, *H. indicus*, *E. prostrata*, *Strychnos nux vomica*, and *A. paniculata* inhibited rattlesnake venom-induced actions (Samy et al., 2008). The animals that received the extract of *Aristolochia odoratissima* leaves orally were prevented against *Bothrops atrox* venom as the mortality of experimental animals reduced from 100% to 80% (Usubillaga et al., 2005). *Tabebuia aurea* decreases hemorrhagic, inflammatory, and myotoxic activities induced by the venom of *Bothrops neuwiedi* (Reis et al., 2014). Ethanolic extract of *Cordia macleodii* bark showed antivenom potential against *Naja venom* (Soni and Bodakhe, 2014). The root extract of *Ophiorrhiza mungos* showed a neutralizing effect against *D. russelii* venom (Krishnan et al., 2014). Extracts of *Euphorbia hirta* and its metabolites protect against snake venom-induced lethality (Gopi et al., 2015).

The methanol root extract of *V. negundo* Linn. and *Emblica officinalis* significantly inhibited the lethal activity induced by *V. russelii* and *N. kaouthia* venom in *in-vivo* studies; *V. russelii* venom-induced hemorrhagic, lethal, dehydrogenating, coagulant and inflammatory activity was significantly inhibited by both plant extracts (Alam and Gomes, 2003). *Hemidesmus indicus* root extracts effectively inhibited viper venom-induced lethal coagulation, hemorrhagic, and inflammatory activities (Alam et al., 1994). Active bioactive metabolites from *S. nux vomica* whole seed extract neutralized lethality, hemorrhage, defibrinogenating PLA2 induced by *D. russelii* venom and enzyme activity, and *N. kaouthia* venom-induced lethality, cardiotoxicity, neurotoxicity, PLA2 enzyme activity and it also neutralized viper venom-induced lipid peroxidation in experimental animals (Chatterjee et al., 2004). Ethanolic root extract from *Cynanchum paniculatum* also shows antivenom properties (Xiong et al., 2018).

## 4 Phytoconstituents with the potential to neutralize snake venom

For many years, it has been well-known that phytoconstituents of numerous plant extracts can neutralize snake venoms. Details of phytoconstituents with snake venom-neutralizing potential are given below: Acids- 2-OH-4 methoxy benzoic acid from *H. indicus* possesses viper venom-induced potent antipyretic and anti-inflammatory properties. It has been investigated that functional groups of these metabolites, particularly hydroxy and methoxy, were partly responsible for neutralizing the hemorrhagic activity and lethal effect of *Vipera russelii* venom (Alam and Gomes, 1998a). The venoms of Indian common snake *V. russelii, N. kaouthia, E. carinatus*, and *Ophiphagus hannah* hemorrhagic, lethal and defibrinogenic action has been neutralized with four metabolites, *Pimpinella anisum* anisic acid, *H. indicus* 2-hydroxy-4-methoxy benzoic acid, *Filipendula ulmaria* salicylic acid and *Salix alba* aspirin in experimental animals. The lethal effect of these snake venoms was neutralized effectively *in vivo* and *in vitro* with anisic acid and 2-hydroxy-4-methoxy benzoic acid. In addition, salicylic acid has effectively neutralized Viper and Echis venom-induced hemorrhagic activity (Alam and Gomes, 1998b). Rosmarinic acid from *Cordia verbenaceae* possesses phospholipase A2 inhibitor activity, and it has been reported as a new antidote against snake *B. jararacussu* venom (Binorkar and Jani, 2012).

Alkaloids- Atropine, in particular members of the Solanaceae family, has an inhibitory function against *Dendroaspis angusticeps* and *D*. *polyposis* venom. These venoms release neurotransmitters at the cholinergic nerve terminals, so it is believed that a cholinergic blocker such as atropine decreases their effects. PLA2 Inhibitor isolated from A. indica methanolic leaf extract neutralizes *R. viper*, *N. naja*, and *N. kaouthia* phospholipase A2 enzymes function in a dose-dependent manner (Mukherjee et al., 2008).

Coumestans and steroids- Beta-sitosterol and stigmasterol isolated from *Pluchea indica* root extract effectively inhibited Viper and cobra venom (Gomes et al., 2007). Beta-sitosterol and stigmasterol have inhibited Venom-induced changes in superoxide dismutase and lipid peroxidation activity. When administered to animals and humans and in *in-vitro* tests, sitosterol shows many pharmacological properties, including anti-inflammatory ability. The capacity of steroids for complex formation has been known in many cases. The physiological importance of these steroids lies in their ability to convert fats and fatty acids into water-soluble or emulsifiable metabolites and thus facilitate intestinal absorption.

Molecules of an extended shape carrying a hydrophilic group at one end can associate with hydrophobic ones surrounding them. The hydrophilic groups turned to the outside and formed molecular complexes with their physicochemical properties. Cholesterol is an occasionally occurring component in plants and has been identified in snake venom antibodies, like onion skins and the root of *Ehretia buxifolia* Roxb. In the early 1900s, cholesterol’s capacity for complex formation became evident, and it was observed that adding cholesterol destroys the violent hemolytic activity of the saponin digitonin. This property of cholesterol explains how cholesterol combines with some of the plasma proteins and interacts with proteins in cell membranes (Yeagle, 1985). Hemolysis is one of the many consequences of the action of snake venoms, phospholipases being the responsible enzymes. These esterases act on the serum lecithin, splitting off the hemolytic lysolecithin. It has been found that cholesterol combines in equimolecular proportion with lysolecithin, the product being devoid of hemolytic activity. Wedelolactone, a coumestan contained in *E. prostrated* L., was reported to be an active metabolite in fighting against snake venoms (Wagner et al., 1986). Wedelolactone, stigmasterol, and sitosterol inhibit the effects of South American rattlesnakes (Mors et al., 1989).

Enzymes, peptides, and pigments-snake venom molecules comprise proteins and some non-protein components. These proteins can be dissolved with natural solvents like bromelain and papain. Bromelain is present in pineapple (*Ananas comosus*), while papain is found in papaya fruit (*Carica papaya*). These two naturally existing proteolytic enzymes can neutralize snake venom proteins. A peptide metabolite (6000 Da) reported anti-cardiotoxic activity against cobra venom was isolated and purified from the plant of *Schummanniophyton magnificum* (Houghton et al., 1992). Turmerin, a protein from turmeric (*Curcuma longa* L.), reported inhibiting the enzymatic activity with neutralization of the edema, cytotoxicity, and myotoxicity of multitoxic phospholipase A2 of cobra (*N. naja*) (Chethankumar and Srinivas, 2008). Melanin isolated from black tea was reported to possess antivenom potential against *Agkistrodon contortrix laticinctus*, *Agkistrodon halys blomhoffii*, and *Crotalus atrox* snake venoms (Hung et al., 2004).

Glycoprotein and glycosides-a multiform glycoprotein with functional oligosaccharides isolated from *M. pruriens* seeds neutralize *E*. *carinatus* venom-induced actions (Guerranti et al., 2004). A glycoprotein (WSG) isolated from *Withania somnifera* is reported to inhibit the phospholipase A2 activity of NN-Xia-PLA2 isolated from cobra venom (*N. naja*), entirely at a mole-to-mole ratio of 1:2 (NN-XIa-PLA2:WSG) (Machiah et al., 2006). It prolonged the death time and reduced the toxicity of the experimental mice approximately ten times as compared to antivenom alone. The WSG also inhibits several other PLA2 isoforms from the venom to a different extent. Hyaluronidase activity induced by cobra (*N. naja*) and viper (*D. russelii*) venoms was inhibited by WSG. It has also been reported to inhibit the hyaluronidase activity of Indian cobra (*N. naja*) venom (Girish et al., 2004; Deepa and Gowda, 2006). Salireposide and benzoylsalireposide isolated from *Symplocos racemosa* showed phosphodiesterase activity against snake venom. The methanolic extract of the stem bark of *S. magnificum* and schumanniofoside, a chromone alkaloidal glycoside, was isolated to reduce black cobra (*Naja melanoleuca*) venom-induced lethal effect in mice. The probable mechanism of this action is oxidative inactivation of the venom.

Phenols- *B. asper* venom-induced PLA2 activity was neutralized by 4- nerolidylcatechol from *Piper peltatum* and *Piper umbellatum* (Nunez et al., 2005). The ethanolic extract of seed kernels from Thai mango (*Mangifera indica* L.) and its major phenolic metabolites pentagalloyl glucopyranose show dose-dependent inhibitory effects on hyaluronidase, phospholipase A2 and L-amino oxidase of *Calloselasma rhodostoma* and *N. kaouthia* venoms in *in-vitro* studies. The anti-hemorrhagic and anti-dermo necrotic activities of seed kernel against both venoms were supported by *in-vivo* studies (Akubue, 1986). The plant polyphenols from the aqueous extracts of *Pentace burmanica*, *Pithecellobium dulce*, *Areca catechu*, and *Quercus infectoria* block nicotinic acetylcholine receptor non-selectively by precipitation of *N. kaouthia* venom (Leanpolchareanchai et al., 2009).

Pterocarpus- Cabenegrin A-1 and Cabenegrin A-2 isolated from an aqueous extract of the root of a South American Plant called Cabeca de Negra have been reported as an anti-snake oral antidote (Nirmal et al., 2008). Similarly, the extract of *Harpalyce Brasilia* Benth, a South American plant commonly called Portuguese snake herb, also yielded cabenegrin A-2 (phenolic pterocarpan in nature). Edunol, a pterocarpan isolated from *Harpalyce brasiliana* used in Brazil against snakebites and roots of *Brongniartia podalyrioides* and *Brongniartia intermedia* (Leguminosae), edunol reduced the expected death rate of mice previously administered with *B. atrox* (Nakagawa et al., 1982) venom along with antiproteolytic, antimyotoxic and PLA two inhibiting properties (Reyes–Chilpa et al., 1992).

Tannins- The tannin from persimmon fruit from *Diospyros kaki* was found to inhibit edema in mice, resulting from envenomation by sea snakes and was reported to improve the survival rate in mice (Okonogi et al., 1979). Ellagic acid, a metabolite isolated from the aqueous extract of *C. sylvestris*, has been reported to have anti-snake venom potential mainly against *Bothrops* genus (daSilva et al., 2008).

Terpenoids-glycyrrhizin, a natural triterpenoid saponin isolated from the root extract of *Glycyrrhiza glabra* (840 Da), has been identified as an inhibitory metabolite against thrombin (Francischetti et al., 1997). This metabolite is well known to possess anti-inflammatory activity, and glycyrrhizin is also reported to show *in-vivo* antithrombic properties against snake venom; it inhibits both *in vitro* and *in-vivo* venom-induced changes in hemostasis, suggesting to possess potential antiophidic activity (Assafim et al., 2006). Potassium salt of gymnemic acid, a triterpenoid glycoside isolated from *G. sylvestre* known to inhibit the ATPase activity in *N.naja* venom (Kini and Gowda, 1982). Lupeol acetate obtained from the Indian sarsaparilla *H. indicus* R.Br. could neutralize the haemorrhage, lethality, defibrinogenation, edema and PLA2 activity induced by *D. russelii* venom significantly. In experimental animals, it also neutralized *N. kaouthia* venom-induced cardiotoxicity, lethality, respiratory changes, and neurotoxicity (Chatterjee et al., 2006). *Bothrops neuwiedi* and *B. jararacussu* venom-induced fibrinogenolytic, hemorrhagic, and caseinolytic activity of class P-1 and three metalloproteases inhibited by neo-clerodane, a diterpenoid, purified from *Baccharis trimera* (Januario et al., 2004). Oleanolic acid inhibited sPLA(2) activities of *N. naja* and *V. Russell* snake venoms in a concentration-dependent manner. Prevention of *in vitro* and *in vivo* sPLA2 activity by oleanolic acid suggests the anti-inflammatory properties of some oleanolic acid-possessing medicinal plants (Dharmappa et al., 2009). The pentacyclic triterpenes are present widely in some anti-snake venom plants like *Centipede minima*, *Aegle marmelos*, *Aloe barbadensis Mill*, *Phyllanthus niruri*, *P. emblica*, *Alstonia scholaris*, *Elephantopus scaber*, etc., showed nearly 20% inhibition against snake venom (Mors et al., 2000). Quinovic acid-3-O-beta-D-fucopyranoside, Quinovic acid-3-O-alpha-rhamnopyranoside (Fatima et al., 2002), and quinovic acid-3-O-beta-D-glucopyranosyl (1-4) beta-D-fucopyranoside obtained from the ethyl acetate extract of *Bridelia ndellensis* barks, and *Mitragyna stipulosa* showed significant neutralizing activity against snake venom phosphodiesterase −1 (Castro et al., 1999; Mostafa et al., 2006). Triterpenoid saponin isolated from *P. macroloba* neutralizes the antiproteolytic and anti-hemorrhagic actions caused by *Bothrops* snake venom. These inhibitors could neutralize the fibrin (ogen) olytic and proteolytic activities of class P-1, P-2 metalloproteases purified from *B. jararacussu* and *B. neuwiedi* venoms (daSilva et al., 2007). Ursolic acid, a common phytoconstituent of several medicinal plants, neutralizes PLA2 enzymes isolated from *N. naja* and *V.* Russell venom (Nataraju et al., 2007). Betulinic, ursolic, and oleanolic also exhibited inhibition against the enzymatic and biological effects induced by a P-I snake venom metalloproteinase (Preciadoa et al., 2018).

Quinonoid Xanthene- Ehretianone, a quinonoid xanthene purified from the root bark of *E. buxifolia*.Rox.B. has been known to have antivenom (*E. carinatus*) activity (Selvanayagam et al., 1996).

Resveratrol–Hong Bei Si Chou is an herbal remedy used to cure snakebites in *China’s Guangxi province*. It was reported that resveratrol (3,4,5-trihydroxy trans-stilbene) purified from the ethyl acetate part of Hong Bei Si Chou could antagonize snake toxins in both *in-vivo* and *in-vitro* conditions (Yang et al., 1998). Alkaloid (12-methoxy-4-methylvoachalotine) extract of *Tabernaemontana catheriensis* neutralized lethality induced by *Crotalus durrissus terrificus* snake venom (Batina et al., 2000).

Other active metabolites and chemical groups-several plant phytoconstituents like flavonoids, xanthenes, polyphenols, quinonoids, and terpenoids have enzyme-inhibiting abilities, protein-binding activity, also neutralize snake venom PLA2 activities of both cobra and viper venom (Alam et al., 1996). Complete inhibition of hemorrhage was observed with the ethanol, ethyl acetate, and aqueous extract of *Bursera simaruba*, *Clusia torresii*, *C. palmana*, *Croton draco*, *Persea americana*, *Phoebe brenesii*, *Pimenta dioica*, *Sapindus saponaria*, *Smilax cuculmeca* and *Virola koschnyi* (Castro et al., 1999). Chemical profiling of these extracts indicates the presence of catequines, anthocyanins, flavones, and condensed tannins, which may be responsible for the inhibitory effect by chelation of the zinc needed for the activity of *B. asper* venoms hemorrhagic metalloproteinases. Plant-derived aristolochic acid, quercetin, indomethacin, tannic acid curcumin, and flavone exhibited inhibition, and aristolochic acid and quercetin completely neutralized the hyaluronidase activity (Pithayanukul et al., 2005). Phytoconstituents of *Andrographis paniculata* exhibited anti-phospholipase A2 activity against the venom of Russell’s viper (*Daboia russellii*) (Shivashankar et al., 2019).

Further, these inhibitors decrease the local tissue damage and reduce the easy diffusion of systemic toxins, thus increasing the survival time. Medicinally important herbal metabolites (acalyphin, chlorogenic acid, stigmasterol, curcumin, and tectoridin) were screened against *Russels viper* PLA2 (Nirmal et al., 2008). These metabolites showed favorable interactions with the amino acid residues at the active site of *R. viper* PLA2, substantiating their proven anti-inflammatory and antidote efficacy. An active metabolite (SNVNF) was purified from the whole seed extract of *S. nux* vomica, which may effectively antagonize *Daboia* Russell venom-induced lethality, defibrinogenating, hemorrhage, PLA2 enzyme activities, edema, and *N. kaouthia* induced lethality, neurotoxicity, cardiotoxicity, and PLA2 enzyme activities. The hexane extract of *C. longa* rhizomes, ar-turmerone (Ferreira et al., 1992), was also reported to inhibit the proliferation of human lymphocytes' natural killer cell activity. This metabolite possesses anti-lethal activity against the venom of *Crotalus durrissimus terificus*. Moreover, when administered in mice, it showed anti-hemorrhagic activity against *B. jararacussu* venom.

## 5 Mechanisms of snake venom neutralization by herbal compounds

Herbal metabolites with snake venom neutralization properties usually follow three mechanisms- 1) venom following herbal metabolites, 2) venom and herbal components in combination, and 3) herbal metabolites following venom. Among these, the third one is close to clinical conditions. One of the key elements for the herbal metabolites to demonstrate their neutralizing actions is the amount of venom. Venom quantity is inversely proportional to the neutralizing effects of herbal metabolites of any plant. So, the venom dose should be tried from a lower to a higher dose. Many hypotheses have been proposed as (1) protein precipitation hypothesis (Vale et al., 2008), (2) enzyme inactivation hypothesis (Hung et al., 2004), (3) chelation hypothesis (Castro et al., 1999), (4) adjuvant activation hypothesis (Alam and Gomes, 1998c), (5) anti-oxidant hypothesis (Chatterjee et al., 2006), (6) protein folding hypothesis, (7) combination hypothesis (Alam and Gomes, 1998a) and many more. The hypothesis mentioned above has its limitations. Among these, the protein-precipitation-inactivation hypothesis is more acceptable. However, more emphasis should be focused on this area soon.

## 6 Benefits and limitations of plant-based remedies

It has been well established that ethnomedicinal plants are pharmacologically efficient against snake venom. The importance of phytochemical compounds obtained from these plants in inhibiting the activity of phospholipase A2, an enzyme frequently present in snake venoms, is highlighted in a study published in the Journal of Pharmacognosy and Phytochemistry (Kankara et al., 2020). Certain ethnomedicinal plants have been shown to prevent snake venom in research studies effectively reported using albino rats. This research finding scientifically justifies the traditional use of these ethnomedicinal plants (Sani et al., 2020). In addition to their ability to neutralize venom, ethnomedical herbs have shown promise in repairing the specific tissue damage caused by snakebites. This implies that the therapeutic potential of these plants is multifaceted and extends beyond the capabilities of conventional antivenoms (Félix-Silva et al., 2017). The usual method for treating snakebites is still traditional antivenom therapies, although ethnomedicinal plants provide a useful alternative. Ethnomedicinal plants are important resources with a wide range of pharmacological properties that make them more accessible and affordable than traditional antivenoms, particularly in areas with limited resources (Deshpande et al., 2022).

Comprehensive scientific research on ethnomedicinal plants demonstrating their efficacy as snakebite antivenom is limited (David Paul Raj et al., 2022). Moreover, the intricate composition of snake venom and its wide-ranging impact on the body make it difficult to create a universally potent antivenom derived from plants. Furthermore, the complex makeup of snake venom and its extensive effects on the body make it challenging to develop a plant-based antivenom for different snake species (Liaqat et al., 2022; Patricia et al., 2022). New treatments are being investigated due to the existing antivenoms' restricted use and stringent storage restrictions. Despite this, there is still an important gap in the literature regarding these herbal remedies' phytochemical composition and safety evaluations (Mokua et al., 2021). Moreover, producing and using plant-based antivenoms is challenging without empirical validation (Omara et al., 2021).

## 7 Future of antivenom and herbal therapy and nanotechnology

Considering the limitations of snake antivenom (Gomes et al., 2010), the world is looking for an alternative to snakebite treatment. Still, no suitable alternative measures, except natural herbal remedies, attract researchers due to their snake antivenom potential. These herbal medicines might be an alternative to cure snake envenomation since they are inexpensive, readily available, stable at room temperature, and can neutralize snake venom components. In the present scenario, the future of snake antivenom lies in herbal compounds, and a combination of these antidotes may be proven as a suitable alternative to snakebites shortly. Combination therapy is a traditional practice of the Ayurvedic system. There are various commercially available therapeutic and ayurvedic medicines, e.g., Articulin-F, which comprises the fixed combination of *C. longa*, *W. somnifera*, *Boswellia serrata*, and zinc for treating osteoarthritis. Trikatu is comprised of long pepper, black pepper, and ginger to treat digestive disorders. In Ayurveda, more than twenty formulations are available, which use a combination of herbal metabolites, metal ions, and spices for practical use. According to Ayurveda, a medicinal plant may need to be administered with other plants to exert its therapeutic effects. The second plant may stimulate the action of the first, whereas the third might help prevent the second plant’s toxicity.

Nanotechnology is an emerging field of science that has revolutionized the progress in chemical, physical, and biological sciences through its applications in biomedical research. Nanotechnology is also contributing to the field of herbal anti-snake venom research. Medication delivery, better stability, reduced toxicity, bioavailability, and targeted drug delivery are all possible benefits of nanotechnology in herbal anti-snake venom. Thus, nanotechnology directly impacts human health and the environment by contributing to the era of biomedical sciences. Due to the miniature size of nanoparticles, these particles can efficiently translocate to their target site through bloodstreams or their entry portals. Antisnake venom herbal metabolites conjugated with nanoparticles are now an expanding area of present-day research. 2-hydroxy-4-methoxy-benzoic acid (HMBA) purified from the root extract of Indian sarsaparilla (*H. indicus*), conjugated with gold nanoparticle when administered, is found to increase the viper venom neutralizing efficacy of HMBA and decreased the toxicity (Saha and Gomes, 2017). Saha et al. have synthesized gold nanoparticles using gold salt, exploiting the reducing property of *Vitex negundo*, and assessed its potentiality against viper venom-induced acute stress and acute cytokine response in the experimental animal model (Saha et al., 2015). This nanoparticle reached the snake venom target sites and neutralized the deleterious venom’s effects on the major organs, including the kidney and liver.

The anti-inflammatory and anti-oxidant potential of curcumin, isolated from *C. longa*, has been reported well (Menon and Sudheer, 2007). When conjugated with gold nanoparticles by the absorption method, curcumin is administered, followed by an assessment of its ant-viper venom activity in the experimental animal model. It neutralizes local damages (Ghosh and Gomes, 2016). It was suggested that curcumin-conjugated gold nanoparticles might act by neutralizing viper venom-induced local damages by inhibiting the pro-oxidant activity of the venom, by direct inhibition at the enzymatic level, and by interfering with cellular markers, including inflammatory markers and anti-oxidants (Ghosh and Gomes, 2016). In *ex-vivo* and *in vitro* research, Oliveira et al. (2019) demonstrated the neutralization of silver nanoparticles against *B. jararacussu* snake venom-induced neurotoxicity and myotoxicity (Soares et al., 2018) have produced an antidote using chitosan polymer against *Bothrops jararaca* and *Bothrops erythromelas* snake venom. They have shown that chitosan polymer nanoparticles have the potential to exhibit immunoadjuvant properties. Nanoparticles have another advantage due to their pharmacokinetics and protein-binding properties, making them a low-cost and readily available alternative for antivenom against snake venom toxins. Cellular signaling molecules, enzymes, cytokines, and pro-inflammatory indicators are the molecular targets for these herb-nano-conjugated biomolecules. However, more profound and thorough research is needed to validate these herbs-nano-conjugates' actions for their approval as an effective alternative antidote against Snakebite (Gomes et al., 2018).

### 7.1 Detection of snake venoms, toxins, and venom antibodies

It might be difficult for victims to determine the snake species that bitten them, and clinical symptoms alone are insufficient to implement an effective treatment strategy. In controlling snake envenomation, the quick or immediate identification of snake venom, including toxin antibodies in bodily fluids, is important. Instantaneous or within short-duration detection of snake venom and venom antibodies in body fluids has a significant role in managing snake envenomation. Bioassays for venom detection have been developed, including immunodiffusion, immunofluorescence, immunoelectrophoresis, haemagglutination, enzyme-linked immunosorbent assay (ELISA), and radioimmunoassay (RIA), among others. So far, ELISA has been extensively used for venom detection and estimation. Snake species diagnosis is challenging because many venomous species are present within the same geographical region, and venom antigen cross-reaction dilutes the results. Lack of specific immunoreagents, lengthy incubation steps, low-level sensitivity, and the need for expensive equipment have restricted the widespread use of routine diagnostic methods such as RIA and ELISA during early 1980. However, significant progress has been made during the last decade to develop species-specific ELISA to detect venom toxins worldwide, particularly in developing countries where snakebite is a major medical and social issue. Species-specific immunoreagents for clinical use have been developed using hybridoma technology and affinity chromatography.

## 8 Statistical study

**FIGURE 1:**
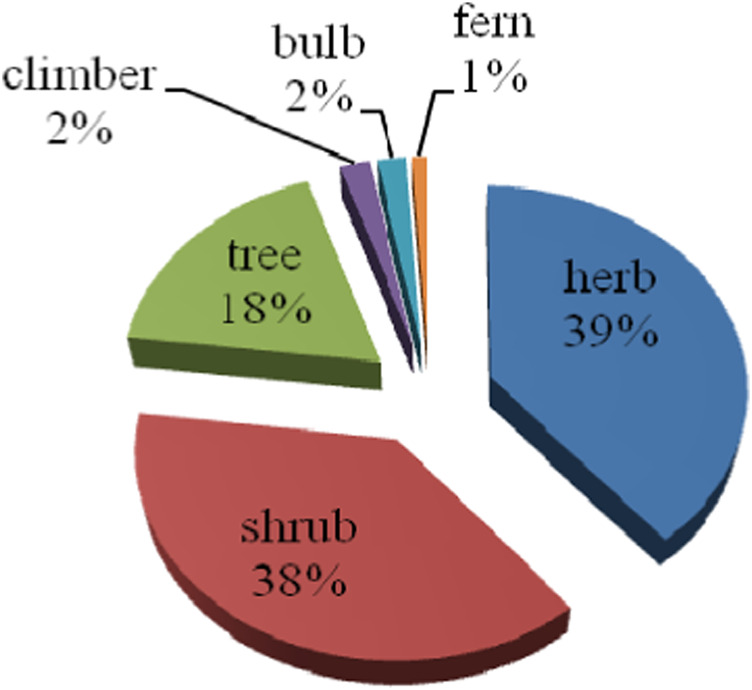
Nature of medicinal plants used in snakebite treatment.

**FIGURE 2:**
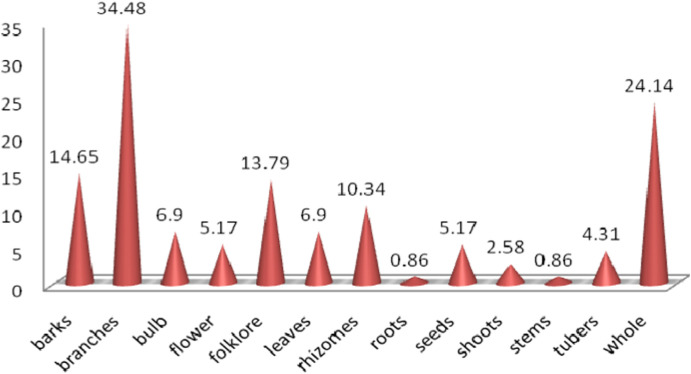
Percentage of medicinal plant parts used in snakebite treatment.

This review aims to gather and organize information on ethnomedicinal plants and their phytoconstituents used for snakebite treatment from various literature sources. Data have represented the plant types, family and parts used, phytoconstituents, etc. This review identifies 116 plant species from 59 families capable of alleviating snakebite. As a result, 39% of herbs, 38% of shrubs, 18% of trees, 2% climbers, 2% bulbs, and 1% of ferns plants have been identified to possess snake antivenom potential (Figure 1). Among these different types of plants, roots (14.65%), leaves (34.48%), rhizomes (6.90%), seeds (5.17%), branches (13.79%), barks (6.90%), stem (10.34%), tuber (0.86%), shoots (5.17%), flower (2.58%), bulb (0.86%), folklore (4.31%) and 28 (24.14%) plants are being used as a whole (Figure 2).

## 9 Conclusion

The most commonly used therapeutic agent for snakebite treatment is the antiophidic serum (antivenom), which is conventionally prepared by injecting a non-lethal dosage of snake venom in mammals like a goat, rabbits, and the horse, is painful for animals and uneconomic. As an alternate option, plants have been used as valuable sources for snakebite treatment because they contain many chemical compounds that can inhibit snake venom toxins. These reviews aimed to give a more thorough understanding of natural inhibitors extracted from plants and used to combat snake venoms and toxins. Additionally, these reviews aimed to enhance our understanding of possible alternatives for snakebite treatment. As reported in this review, the large, diverse, pharmacologically active molecules in medicinal plant extracts make them an attractive candidate for the future discovery of snake antivenom compounds for snakebite treatment. Nevertheless, an adequate understanding of snake-venom neutralization mechanisms requires understanding the structure and chemical nature of snake venom and plant metabolites. Interactions between snake venoms and some plant constituents have been described. However, candidate molecules responsible for snake venom neutralization and their mechanism function have yet to be characterized and explained in many plant species. These findings need additional investigation in animal preclinical trials to ensure future human clinical applications. In conclusion, this review has confirmed the ethnomedical use of 116 medicinal plants for treating snakebite victims. Besides, a more comprehensive analysis is required to validate the effectiveness of plant bioactive substances and their mechanism against snake venoms of different geographical origins and to develop nano-herbal- conjugates to save thousands of human deaths every year worldwide.

## Abbreviations

BthTX1, Bothropstoxin-1; BthTX2, Bothropstoxin-2; ELISA, Eenzyme Linked Immunosorbent Assay; IgG, Immunoglobulin G; PLA2, Phospholipase A2; RIA, Radioimmunoassay; WHO, World Health Organisation.
