# Supplementary material for: A comprehensive review on recent advances in the use of ethnomedicinal plants and their metabolites in snake bite treatment
Source: Front Pharmacol. 2025 Mar 12;16:1548929. doi: 10.3389/fphar.2025.1548929 (PMC11973492; doi:10.3389/fphar.2025.1548929)
Supplement: Supplementary file 1 [file Table1.doc]

**Table 1 List of ethnomedicinal plants and their parts used for the treatment of snakebites**

| **S. No.** | **Plant name** | **Family** | **Plant part (s) used** | **Reference** |
| --- | --- | --- | --- | --- |
|  | *Guiera senegalensis* **J.F.Gmel.** | Combretaceae | Leaves | Abubakar , 2000 |
|  | *Achyranthes aspera* L. | Amaranthaceae | Whole Plant | Ahmad, 2007; Gomes et al., 2010; Makhija and Khamar, 2010 |
|  | *Emblica officinalis* Geartn. | Phyllanthaceae | Roots | Alam and Gomes, 2003 |
|  | *Vitex negundo* Linn. | Lemiaceae | Roots |
|  | *Hemidesmus indicus* [(L.) R. Br. ex Schult.](http://www.theplantlist.org/tpl1.1/record/tro-2609794) | Apocynaceae | Roots | Alam et al., 2003 |
|  | *Annona senegalensis* Pers. | Annonaceae | Root and bark | Adzu et al., 2005 |
|  | *Musa paradiasica* L. | Musaceae | Juices | Borges et al., 2005; Gomes et al., 2010, Makhija and Khamar, 2010 |
|  | *Strychnos nux-vomica* L. | Loganiaceae | Seeds | Chatterjee et al., 2004 |
|  | *Galactia glauscescens* [var. *obtusa* (Benth.) Burkart](http://www.theplantlist.org/tpl1.1/record/ild-10810) | Fabaceae | Leaves | Dalbelo et al., 2008 |
|  | *Mikania glomerata* [var. *glomerata*](http://www.theplantlist.org/tpl1.1/record/gcc-146802) | Asteraceae | Root, stem, and leaves | de Silva et al., 2005 |
|  | *Croton urucurana* Baill. | Euphorbiaceae | Whole Plant | Esmeraldino et al., 2005; Gomes et al., 2010 |
|  | *Acorus calamus* Linn. | Acoraceae | Rhizomes | Gomes et al., 2010; Makhija and Khamar, 2010 |
|  | *Calycopteris floribunda* Roxb. | Combretaceae | Leaves | Gomes et al., 2010; Shekhar et al., 2011 |
|  | *Cassia occidentalis* Linn. | Fabaceae | Roots | Gomes et al., 2010; Shekhar et al., 2011; Ahmad, 2007 |
|  | *Abrus precatorious* L. | Fabaceae | Roots | Gomes et al., 2010 |
|  | *Antidesma bunius* Linn. | Phyllanthaceae | Leaves |
|  | *Arctium lappa* L. | Asteraceae | Whole Plant |
|  | *Actaea racemosa* L. | Ranunculaceae | Rhizomes |
|  | *Aloe barbadensis* Mill | Asphodelaceae | Leaves |
|  | *Buchnania lanzan* Sp | Anacardiaceae | Stem and bark |
|  | *Calotropis gigantia* [(L.) R. Br. ex Schult.](http://www.theplantlist.org/tpl1.1/record/tro-50183675) | Apocynaceae | Leaves and roots |
|  | *Biophytum sensitivum* [var. *assamicum* Edgew. & Hook. F.](http://www.theplantlist.org/tpl1.1/record/kew-2676193) | Oxalidaceae | Whole Plant |
|  | *Cecropia peltata* L. | Utricaceae | Leaves |
|  | *Cucumis colocynthis* L. | Cucurbitaceae | Roots |
|  | *Columnea kalbreyeriana* Mast. | Gensneriaceae | Whole Plant |
|  | *Bombax ceiba* Linn. | Malvaceae | Shoot and leaves |
|  | *Diodia scandens* Sw. | Rubiaceae | Whole plant |
|  | *Echinacea pallid* [(Nutt.) Nutt.](http://www.theplantlist.org/tpl1.1/record/gcc-119485) | Asteraceae | Whole Plant |
|  | *Enicostemma axillare* **(Lam.) Raynal** | Gentianaceae | Leaves |
|  | *Equisetum geganteum* L. | Equisetaceae | Rhizomes |
|  | *Ficus lacor* [Buch.-Ham.](http://www.theplantlist.org/tpl1.1/record/kew-2810991) | Moraceae | Latex |
|  | *Ficus religiosa* L. | Moraceae | Flower |
|  | *Impatiens balsamina* L. | Balsaminacae | Flower |
|  | *Leucas linifolia* [(Roth) Spreng.](http://www.theplantlist.org/tpl1.1/record/kew-111767) | Lamiaceae | Whole Plant |
|  | *Lysimachia numumularia* L. | Primulaceae | Whole Plant |
|  | *Mandevilla illustris* [(Vell.) Woodson](http://www.theplantlist.org/tpl1.1/record/kew-119281) | Apocynaceae | Whole Plant |
|  | *Melianthus major* L. | Francoaceae | Flower |
|  | *Psolarea corylifolia* L. | Fabaceae | Seeds |  |
|  | *Raphanus sativus* L. | Columbidae | Tuber |
|  | *Oldenlandia corymbosa* L. | Rubiaceae | Whole Plant |
|  | *Nerium indicum* Mill | Apocynaceae | Leaves |
|  | *Papaver somniferum* L. | Papaveraceae | Whole Plant |
|  | *Paris polyphylla* Sm. | Melanthiaceae | Roots |
|  | *Passiflora quadrangularis* L. | Passifloaceae | Leaves and branches |
|  | *Selaginella articulate* [(Kunze) Spring](http://www.theplantlist.org/tpl1.1/record/tro-26610849) | Sellaginaceae | Whole Plant |
|  | [*Senna* *dariensis*](http://www.theplantlist.org/tpl1.1/record/ild-21330) [(Britton & Rose) H.S.Irwin & Ba](http://www.theplantlist.org/tpl1.1/record/ild-21330) | Fabaceae | Whole Plant |
|  | *Origanum dictamnus* L. | Lamiaceae | Seeds |
|  | *Tabernaemontana catheriensis* [A.DC.](http://www.theplantlist.org/tpl1.1/record/kew-200611) | Apocynaceae | Whole plant extract |
|  | *Woofordia fruitcosa* [(L.) Kurz](https://mpns.science.kew.org/mpns-portal/plantDetail?plantId=465124&query=Woofordia+fruitcosa&filter=&fuzzy=true&nameType=all&dbs=wcsCmp) | Lythraceae | Whole Plant |
|  | [*Xanthium* *sibiricum* Patrin ex Widder](http://www.theplantlist.org/tpl1.1/record/gcc-9198) | Asteraceae | Leaves |
|  | *Ulmus rubra* Muhl. | Ulmaceae | Leaves |
|  | *Moringa oleifera* Lamk | Moringaceae | Shoot and leaves | Gomes et al., 2010; Makhija and Khamar, 2010 |
|  | *Ophiorrhiza mango* D.C. | Rubiaceae | Roots |
|  | *Rauwolfia serpentine* (Benth) | Apocynaeae | Whole Plant |
|  | *Mucuna pruriens var.utilis* | Fabaceae | Seeds and leaves | Houghton and Skari, 1994 |
|  | *Schumanniophyton magnificum* [(K.Schum.) Harms](http://www.theplantlist.org/tpl1.1/record/kew-188461) | Rubiaceae | Bark |
|  | *Strophanthus hispidus* D.C. | Apocynaceae | Leaves |
|  | *Strophanthus gratus* [(Wall. & Hook.) Baill.](http://www.theplantlist.org/tpl1.1/record/kew-198229) | Apocynaceae | Leaves |
|  | *Casearia mariquitensis* Kunth | Orchidaceae | Stems | Izidoro, 2003 |
|  | *Alangium salvifolium* Merr. | Cornaceae | Roots | Martz, 1992; Gomes et al., 2010; Shekhar et al., 2011 |
|  | *Andrographis paniculata* (Burm.f.) Nees | Acanthaceae | Whole Plant | Meenatchisundaram et al., 2009; Makhija and Kamar, 2010; John et al., 2011 |
|  | *Diospyros kaki* L.f. | Ebenaceae | Folkfore | Martz et al., 1992; Gomes et al., 2010 |
|  | *Mimosa pudica* L. | Fabaceae | Roots and whole plant | Mahanta, 2001 |
|  | *Echinacea angustifolia* D.C. | Asteraceae | Roots and rhizomes | Lobo et al., 2006; Gomes et al., 2010 |
|  | *Gymnema sylvestre* [(Retz.) R.Br. ex Sm.](http://www.theplantlist.org/tpl1.1/record/kew-2835456) | Apocynaceae | Roots |
|  | *Echinacea purpurea* [(L.) Moench](http://www.theplantlist.org/tpl1.1/record/gcc-135149) | Asteraceae | Extracts of whole Plant | Lobo et al., 2006; Chaves et al., 2007; Gomes et al., 2010 |
|  | *Tamarindus indica L.* | Fabaceae | Seeds | Lobo et al., 2006; Ushanandini et al., 2006; Gomes et al., 2010 |
|  | *Aristolochia shimadai* Duch. | Aristolochiaceae | Folkfore | Martz, 1992; Gomes, 2010 |
|  | *Vitis vinifera* L. | Vitaceae | Seeds | Mahadeswaraswamy et al., 2009 |
|  | *Artemisia campestris* L. | Asteraceae | Leaves | Memmi et al., 2007 |
|  | *Azadirachta indica* A.Juss. | Meliaceae | Leaves | Mukherjee et al., 2008; Gomes et al., 2010; Makhija and Khamar, 2010 |
|  | *Rhizoma paridis* (Chonglou) | Liliaceae | Roots | Mendes et al., 2008; Gomes et al., 2010 |
|  | *Schizolobium parahyba* **(Vell.) S.F.Blake** | Fabaceae | Leaves | Mendes, 2008; Vale, 2008 |
|  | *Tabebuia avellanedae* [Lorentz ex Griseb.](http://www.theplantlist.org/tpl1.1/record/kew-318725) | Bignoniaceae | Bark | Nishijima et al., 2009; Gomes et al., 2010 |
|  | *Crinum jagus* [J.Thomps.](http://www.theplantlist.org/tpl1.1/record/kew-298748) | Amaryllaceae | Bulb | Ode, 2006; Asuzu, 2003 |
|  | *Dracontium croatii* G.H. Zhu | Araceae | Rhizomes | Otero et al., 2000; Gomes et al., 2010 |
|  | *Costus lasius* Loes. | Costaceae | Leaves, branches, and stem |
|  | *Sida acuta* [Burm.f.](http://www.theplantlist.org/tpl1.1/record/kew-2588280) | Malvaceae | Whole plant |
|  | *Aristolochia grandiflora* Sw*.* | Aristolochiaceae | Whole plant | Otero et al., 2000 |
|  | *Allamanda cathartica* L. | Apocynaceae | Leaves, branches, and stems |
|  | *Bixa orellana* L. | Bixaceae | Leaves, branches, and shoot |
|  | *Brownea rosa* Pers. | Fabaceae | Stem and barks |
|  | *Castilla elastic* Cerv. | Moraceae | Leaves, branches, and stem |
|  | *Citrus limon* [(L.) Osbeck](http://www.theplantlist.org/tpl1.1/record/tro-28101295) | Rutaceae | Fruit |
|  | *Capsicum frutescens* L. | Solanaceae | Fruits |
|  | *Ficus nymphaeifolia* Mill. | Moraceae | Leaves, branches, and shoot |
|  | *Crescentia cujete* L. | Bignoniaceae | Fruit |
|  | *Gonzalagunia panamensis* [(Cav.) K. Schum.](http://www.theplantlist.org/tpl1.1/record/kew-91700) | Rubiaceae | Leaves and branches |
|  | *Heliconia curtispatha* **Petersen** | Heliconiaceae | Rhizomes |
|  | *Hyptis capitata* Jacq. | Lamiaceae | Leaves, branches, and stem |
|  | *Ipomoea cairica* [(L.) Sweet](http://www.theplantlist.org/tpl1.1/record/tro-8501793) | Convolvulaceae | Leaves, branches and stem |
|  | *Neurolaena* lobata (L.) R.Br.ex Cass | Asteraceae | Leaves, branches and stem |
|  | *Philodendron tripartitum* [(Jacq.) Schott](http://www.theplantlist.org/tpl1.1/record/kew-152003) | Araceae | Shoot and leaves |
|  | *Piper arboreum* Aubl. | Poperaceae | Leaves and branches |
|  | *Piper pulchrum* C. DC. | Piperaceae | Leaves, branches and stem |
|  | *Pleopeltis percussa* **(Cav.) Hook. & Grev.** | Polypediaceae | Whole Plant |
|  | *Pseudelephantopus spicatus* [(B.Juss. ex Aubl.) Rohr ex C.F.Baker](http://www.theplantlist.org/tpl1.1/record/gcc-148626) | Asteraceae | Whole Plant |
|  | *Renealmia alpinia* **(Rottb.) Maas** | Zingerbiaceae | Rhizomes |
|  | *Trichomanes elegans* Rich. | Hymenophyllaceae | Whole Plant |  |
|  | *Strychnos xinguensis* **Krukoff** | Loganiaceae | Stem, leaves, and branches |
|  | *Struthanthus orbicularis* [(Kunth) Eichler](http://www.theplantlist.org/tpl1.1/record/kew-2595336) | Loranthaceae | Leaves, branches, and shoot |
|  | *Siparuna thecaphora* **(Poepp. & Endl.) A.DC.** | Siparunaceae | Leaves, stems, and branches |
|  | *Tabebuia rosea* **(Bertol.) Bertero ex A.DC.** | Bignoniaceae | Stem barks |
|  | *Eclipta prostrata* L. | Asteraceae | Whole plant | Pithayanukul et al., 2004; Gomes et al., 2010 |
|  | *Casearia sylvestris* [var. *angustifolia* Uittien](http://www.theplantlist.org/tpl1.1/record/kew-4702407) | Saliaceae | Leaves and bark | Raslan, 2002 |
|  | *Curcuma longa* L. | Zingerberaceae | Rhizomes | Ratranabanangkoon et al., 1993 |
|  | *Cinnamomum zeylanicum* Blume | Lauraceae | Whole Plant | Ruppelt et al., 1991 |
|  | *Marsypianthes chamaedrys* **(Vahl) Kuntze** | Lamiaceae | Whole Plant | Ruppelt et al., 1991; Gomes et al., 2010 |
|  | *Acalypha indica* L. | Euphorbiaceae | Roots | Shirwaikar et al., 2004 |
|  | *Cordia verbenaceae* A.D.C. | Boraginaceae | Rosmarinic acid extract | Ticli et al., 2005; Gomes et al., 2010 |
|  | *Aristolochia odaratissima* L. | Aristolochiaceae | Leaves | Usubillaga et al., 2005 |
|  | *Nerium oleander* L. | Apocynaceae | Leaves | Upasini et al., 2017 |

**Table 2 List of phytoconstituents and plant sources used for the treatment of snakebites**

**Acids**

| **Compounds** | **Plant sources** | **Anti-snake venom activities** | **Snake species** | **References** |
| --- | --- | --- | --- | --- |
| 2-OH-4-methoxy benzoic acid | [*Hemidesmus* *indicus* (L.) R. Br. ex Schult.](http://www.theplantlist.org/tpl1.1/record/tro-2609794) | Antiserum activity, Anti-pyretic, Anti- hemorrhagic, Anti-inflammatory, Anti-lethal, and Anti-oxidant activity. | Viper species, *Vipera russelii* | Alam and Gomes, 1998 |
| Anisic Acid | *Pimpinella anisum* L*.* | Anti-lethal activity | Some Species |
| Rosmarinic Acid | *Cordia verbenaceae* [I.M.Johnst.](http://www.theplantlist.org/tpl1.1/record/kew-2736515) | Anti- PLA2 activity | *Bothrops jararacussu* | Binorkar et al., 2012 |
| Salicylic acid, aspirin | *Salix alba* L. | Antivenom activity | Viper and Echis snake species | Gomes et al., 2010 |
| Aristolochic acid | *Aristolochia* sp. | Anti- PLA, Anti- lytic and Anti- edematose properties | Some snake species | Wagner and Proksch, 1985 |

**Alkaloids**

| **Compounds** | **Plant sources** | **Anti-snake venom activities** | **Snake species** | **References** |
| --- | --- | --- | --- | --- |
| Atropine | Solanaceae family | Antivenom activities | *Green mamba* and *Black mamba* (*Dendroaspis polylepsis and D. angusticeps* ) | Gomes et al., 2010 |
| AIPLAI (*Azadirachta indica* PLA2 Inhibitor) | *Azadirachta indica* [A.Juss.](http://www.theplantlist.org/tpl1.1/record/kew-2667002) | Anti- PLA2 activity | Cobra species, Russel's viper, *Naja naja*, *N. kaouthia*, *Daboia russeli* | Mukherjee et al., 2008 |

**Coumestans and Steroids**

| **Compounds** | **Plant sources** | **Anti-snake venom activities** | **Snake species** | **References** |
| --- | --- | --- | --- | --- |
| Beta sitosterol and Sitosterol | *Pluchea indica* [(L.) Less.](http://www.theplantlist.org/tpl1.1/record/gcc-34323) | Anti-lipid peroxidation, Anti- superoxide dismutase activity, Anti-inflammatory activity | Viper and Cobra species | Mors, 1989; Gomes et al., 2007 |
| Wedelolactone | *Eclipta prostrata* L. | Antivenom activity | South American rattlesnake | Wagner et al, 1986; Mors et al., 1989 |

**Enzymes, peptides, and pigments**

| **Compounds** | **Plant sources** | **Anti-snake venom activities** | **Snake species** | **References** |
| --- | --- | --- | --- | --- |
| Turmeric | *Curcuma longa* L. | Anti- enzymatic, Anti- cytotoxic, Anti-myotoxic, Anti- PLA2 activity | Some snake species | Chethankumar and Srinivas, 2008 |
| Bromelain | *Ananas comosus* [(L.) Merr.](http://www.theplantlist.org/tpl1.1/record/kew-219669) | Antivenom activity | Some snake species | Gomes et al., 2010 |
| Papain | [*Papaya* *carica* Gaertn.](http://www.theplantlist.org/tpl1.1/record/tro-6100073) | Antivenom activity | Some snake species |
| Peptide | *Schumanniophyton magnificum* [(K.Schum.) Harms](http://www.theplantlist.org/tpl1.1/record/kew-188461) | Anti-cardiotoxicity | Some snake species | Houghton et al., 1992 |
| Melanin | [*Camellia* *sinensis* (L.) Kuntze](http://www.theplantlist.org/tpl1.1/record/kew-2694880) | Antivenom activity | *Agkistrodon contortrix laticinctus*, *A. halys blomhoffi*, *Crotalus atrox* | Hung et al., 2004 |

**Glycoproteins and Glycosides**

| **Compounds** | **Plant sources** | **Anti-snake venom activities** | **Snake species** | **References** |
| --- | --- | --- | --- | --- |
| Benzoylsalireposide and Salireposide | *Symplocos racemosa* **Roxb** | Anti- Phosphodiesterases 1 activity | Some snake species | Girish and Kemparaju, 2005 |
| Glycoprotein | *Mucuna pruriens* [(L.) D.C.](http://www.theplantlist.org/tpl1.1/record/ild-2863) | Antivenom activity | *Echis carinatus* | Guerranti et al., 2004 |
| WSG | *Withania somnifera* **(L.) Dunal** | Anti- PLA2 activity, Anti- hyaluronidase activity | Cobra species, *Naja naja*, *Daboia russeli* | Machiah et al., 2006; Deepa and Gowda, 2006 |

**Phenols**

| **Compounds** | **Plant sources** | **Anti-snake venom activities** | **Snake species** | **References** |
| --- | --- | --- | --- | --- |
| Pentagalloyl glucopyranose | *Mangifera indica* L. | Anti-hemorrhagic, Anti-dermonecrotic, Anti- hyaluronidase activity, Anti- PLA2 activity, Anti- L- amino oxidase activity | *Calloselasma rhodostoma*, *Naja naja* and *Naja kaouthia* | Akubue, 1986 |
| Polyphenols | *Pentace burmanica* **Kurz**, *Pithecellobium dulce* **(Roxb.) Benth.***, Areca catechu* L., *Quercus infectoria* [**G.Olivier**](http://www.theplantlist.org/tpl1.1/record/kew-173331) | Blocks Nicotinic acetylcholine receptor | *Naja naja* and *Naja kaouthia* | Leanpochareenchai et al., 2009 |
| 4-nerolidylcatechol | *Piper umbellatem* L., *Piper peltatum* L. | Anti- PLA2 activity | Some snake species | Nunez et al., 2005 |

**Pterocarpens**

| **Compounds** | **Plant sources** | **Anti-snake venom activities** | **Snake species** | **References** |
| --- | --- | --- | --- | --- |
| Edunol | [*Harpalyce* *acunae* Borhidi & O.Muniz](http://www.theplantlist.org/tpl1.1/record/ild-21117) | Anti-proteolytic, Anti- myotoxic, and Anti- PLA2 activity | *Bothrops atrox* | Nakagawa et al., 1982; Reyes-Chilpa et al., 1994 |
| Cabenegrin A-1 and Cabenegrin A-2 | [*Annona* *purpurea* Moc. & Sessé ex Dunal](https://en.wikipedia.org/wiki/Annona_purpurea) | Antivenom activity | Some snake species | Nirmal et al., 2008 |

**Tannins**

| **Compounds** | **Plant sources** | **Anti-snake venom activity** | **Snake species** | **References** |
| --- | --- | --- | --- | --- |
| Ellagic acid | *Casearia sylvestris* Sw. | Antivenom activity | Bothrops genus | da Silva et al., 2008 |
| Tannin | [*Diospyros* *kaki* L.f.](http://www.theplantlist.org/tpl1.1/record/kew-2769959) | Anti-edema activity | Some snake species | Okonogi et al., 1979 |

**Terpenoids**

| **Compounds** | **Plant sources** | **Anti-snake venom activity** | **Snake species** | **References** |
| --- | --- | --- | --- | --- |
| Quinovic acid-3-O-beta-D-fucopyranoside and quinovic acid-3-O-beta-D-lucopyranosyl  (1-4)-beta-D-fucopyranoside | [*Bridelia* *ndellensis* Beille](http://www.theplantlist.org/tpl1.1/record/kew-24581)*, Mitragyna stipulosa* | Anti- Phosphodiesteraseses activity | Some snake species | Castro et al., 1999; Mostafa et al., 2006 |
| Lupeol acetate | *Hemidesmus indicus* R.Br. | Anti-PLA2 activity, Anti-lethal, Anti-hemorrhagic activity, Anti- cardiotoxicity, Anti-lethality, and Anti-neurotoxicity | *Daboia russeli, Naja kaouthia* | Chatterjee et al., 2006 |
| Triterpenoid Saponin | *Pentaclethra macroloba* **(Willd.) Kuntze** | Anti-proteolytic activity, Anti-hemorrhagic activity, Anti-fibrigenolytic activity | Bothrops species | da Silva et al., 2007 |
| Glycyrrhizin | *Glycyrrhiza glabra* L. | Anti-thrombic properties, Anti-inflammatory activity | Some snake species | Francischetti et al., 1997; Assafim et al., 2006 |
| Neo-clerodane | *Briccharis trimera* [(Less.) DC.](http://www.theplantlist.org/tpl1.1/record/gcc-111448) | Anti-fibrigenolytic activity, Anti-hemorrhagic, Anti-caseinolytic activity | *Bothrops neuwiedi*, *B. jararacussu* | Januario et al., 2004 |
| Potassium salt of gymnastic acid | *Gymnema sylvestris* Wall*.* | Anti- ATPase activity | *Naja naja* | Kini and Gowda, 1982 |
| Pentacyclic triterpenes | *Aegle marmelos* [**(L.) Correa**](http://www.theplantlist.org/tpl1.1/record/kew-2623456)*, Centipeda minima* [**(L.) A.Braun & Asch.**](http://www.theplantlist.org/tpl1.1/record/gcc-75376)*, Aloe barbadensis* Mill.*, Phyllanthus niruri* L. *, Alstonia scholaris* **(L.) R. Br.***, Phyllanthus emblica* L., *Elephantopus scaber* L. | Antivenom activity | Some snake species | Mors et al., 2000 |
| Ursolic acid | [*Eriobotrya* *japonica* (Thunb.) Lindl.](http://www.theplantlist.org/tpl1.1/record/rjp-1354) | Anti- PLA2 activity | *Naja naja*, *Vipera russeli* | Nataraju et al., 2007 |

**Qunonoid Xanthene**

| **Compound** | **Plant sources** | **Anti-snake venom activity** | **Snake species** | **References** |
| --- | --- | --- | --- | --- |
| Ehretianone | *Ehretia buxifolia* Rox B, | Antivenom activity | *Echis carinatus* | Selvanayagam et al., 1996 |

**Resveratrol**

| **Compounds** | **Plant sources** | **Anti-snake venom activity** | **Snake species** | **References** |
| --- | --- | --- | --- | --- |
| Alkaloid (12-methoxy-methylvoachalotine) | *Tabernaemontana catharinensis* A.DC. | Antivenom activity | *Crotalus durissus terrificus* | Batina Mde et al., 2000 |
| Resveratrol (3,4,5,-trihydroxytransstilbene) | *Cissus assamica* **(M.A.Lawson) Craib** | Antivenom activity | Some snake species | Yang et al., 1998 |

Miscellaneous chemical groups and compounds

| **Chemical groups and compounds** | **Plant sources** | **Anti-snake venom activity** | **Snake species** | **References** |
| --- | --- | --- | --- | --- |
| Flavonoid, quinonoid, xanthenes,  Polyphenols and terpenoids | [***Bursera* *simaruba* (L.) Sarg.**](http://www.theplantlist.org/tpl1.1/record/kew-2687649)*,****Clusia* *torresii* Standl.***,* ***Clusia* *palmana* Standl.***,* [***Croton* *draco* Schltdl.**](http://www.theplantlist.org/tpl1.1/record/kew-49807)*,* [***Persea* *americana* Mill.**](http://www.theplantlist.org/tpl1.1/record/kew-2529835)*,* ***Phoebe* *brenesii* Standl.***,* [***Pimenta* *dioica* (L.) Merr.**](http://www.theplantlist.org/tpl1.1/record/kew-156136)*,* [***Sapindus* *saponaria* L.**](http://www.theplantlist.org/tpl1.1/record/kew-2582258)*,* and***Virola* *koschnyi* Warb.** | Anti- PLA2 activity | Viper and Cobra species | Alam et al., 1996 |
| SNVNF | [*Strychnos* *nux-vomica* L.](http://www.theplantlist.org/tpl1.1/record/kew-2598138) | Anti-lethal, Anti-hemorrhagic, Anti-PLA2 activity | Some snake species | Ferreira et al., 1992 |
| Curcumin and tectoridin | [*Curcuma* *longa* L.](http://www.theplantlist.org/tpl1.1/record/kew-235249) | Antivenom activity | Some snake species | Gomes et al., 2010 |
| Ar- turmerone | [*Curcuma* *longa* L.](http://www.theplantlist.org/tpl1.1/record/kew-235249) | Inhibits proliferation of natural killer cells and human lymphocytes, Antilethal activity | *Crotalus durrisus terrificus*, *Bothrops jararacussu* | Hung et al., 2004 |
| Acalyphin, chlorogenic acid, stigmasterol | [*Lonicera* *japonica* Thunb.](http://www.theplantlist.org/tpl1.1/record/kew-2339716)*, Hemidesmus indicus* R.Br. | Anti- PLA2 activity | *Russels viper* | Nirmal et al., 2008 |

**Table 3 Antiophidian mechanisms**

| **Compound** | **Antiophidian mechanisms** | **References** |
| --- | --- | --- |
| Tannins | Anti-lethal activity | Abubakar et al., 2000 |
| Benzoylsalireposide and Salireposide | Anti-Phosphodiesterase 1 activity | Ahmad et al., 2003 |
| 2-hydroxy-4-methoxy benzoic acid | Anti-lethal activity, anti-hemorrhagic activity, coagulant, defibrinogenating agent, fibrinolytic activity | Alam et al., 1996 |
| Amide | Anti-lethal activity, Anti-hemorrhagic, defibrinating, Anti PLA2 activity | Chatterjee et al., 2004 |
| Edunol | Anti-lethal activity | Chilpa, 1994; Mors, 2000 |
| Clerodane diterpenoid | Anti-proteolytic and Anti-hemorrhagic properties | Januario et al., 2004 |
| Sitosterol | Anti-proteolytic, anti-hyaluronidase activity, anti-lethality, anti-myotoxic activity | Mahanta et al., 2001; Girish et al., 2004 |
| Anisodamine | Cholinergic receptor-blocking agents | Makhija and Khamar, 2010 |
| Betulin and betulin acid | Anti-PLA2 activity |
| Steroids | Anti-PLA2 activity |
| Caffeic acid and derivatives | Antidotes |
| Wedelolactone | Anti-myotoxic and anti-hemorrhagic activity | Mors et al., 1989; Melo et al., 1994 |
| 4-nerolidylcatechol | Anti-PLA2 activity, Anti-myotoxic | Nunez et al., 2005 |
| Flavonoids | Hemorrhagic activity | Pereira et al., 1991 |
| Ehretianone | Anti-lethal activity | Selvanayagam et al., 1996 |
| Rosmarinic acid | Anti-PLA2 activity | Ticli et al., 2005 |
| Aristolochic acid | Anti PLA2 activity | Vishwanath et al., 1987 |

**References**

Abubakar, M.S., Sule, M.I., Pateh, U.U., Abdurahman, E .M., Haruna, A.K., Jahun, B.M., 2000. *In vitro* snake venom detoxifying action of the leaf extract of *Guiera senegalensis*. *Journal of Ethnopharmacology* 69 (3), 253-257.

Ahmad, S.S., 2007. Medicinal wild plants from Lahore-Islamabad Motorway. Pakistan Journal of Botany 39 (2), 355-375.

Akubue, P.I., 1986. Schumanniofoside, the antisnake venom principle from the stem bark of *Schumanniohyton magnificum* Harms. Journal of Ethnopharmacology 18(2), 167-172.

Alam, M.I., Auddy, B., Gomes, A., 1996.Viper venom neutralization by Indian medicinal plant (*Hemidesmus indicus* and *Pluchea indica*) root extracts. *Phytotherapy Research* 10 (1), 58-61.

Alam, M.I., Gomes, A., 2003. Snake venom neutralization by Indian medicinal plants (*Vitex negundo* and *Emblica officinalis*) root extracts. *Journal of Ethnopharmacology 86* (1), 75-80. https://[doi.org/10.1016/s0378-8741(03)00049-7](https://doi.org/10.1016/s0378-8741(03)00049-7)

Assafim, M., Ferreira, M.S., Frattani, F.S., Guimaraes, J.A, Monteiro, R.Q., Zingali, R.B., 2006. Counteracting effect of glycyrrhizin on the hemostatic abnormalities induced by *Bothrops jararaca* snake venom *Br. Journal of Pharmacology* 148 (6), 807-813.

Asuzu, I.U., Harvey, A.L., 2003. The antisnake venom activities of *Parkia biglobosa* (Mimosaceae) stem bark extract. *Toxicon* 42 (7), 763-768.

Batina, Mde, F.,Cintra, A.C., Veronese, E.L., Lavrador, M.A., Giglio, J.R., Pereira, P.S., Dias, D.A., Franca, S.C., Sampaio, S.V., 2000. Inhibition of the lethal and myotoxic activities of *Crotallus durissus* terrificus venom by *Taberenaemontana catherinensis;* Identification of one of the active components. *Planta Medica* 66 (5), 424-428.

Binorkar, S.V., Jani, D.K., 2012. Profile of Medicinal plants with anti-ophidian property. *Journal of Pharmacy and Scientific Innovation* 1 (5), 13-20.

Borges, M.H., Alves, D.L.F., Raslan, D.S., Pilo-veleso, D., Rodrigues, V.M., Homsi-Br,eburgo, M.I., de Lima, M.E., 2005. Neutralizing properties of *Musa paradisiaca* L. (Musaceae) juice on Phospholipase A2, myotoxic, hemorrhagic and lethal activities of crotalidae venoms. Journal of Ethnopharmacology 98 (1-2), 21-29.

Castro, O., Gutierrez, J.M., Barrios, M., Castro, I., Romero, M., and Umana, E., 1999. Neutralization of the hemorrhagic effect induced by *Bothrops asper* (Serpentes:Viperadae) venom with tropical plant extracts. *Revista de Biologia Tropical* 47(3), 605-616.

Chatterjee, I., Chakravarthy, A.K., Gomes, A., 2006. *Daboia russeli* and *Naja Kaouthia* venom neutralization by lupeol acetate isolated from the root extract of Indian sarsaparilla *Hemidesmus indicus* R.Br. *Journal of Ethnopharmacology* 106 (1), 38-43.

Chatterjee, I., Chakravarty, A.K., Gomes, A., 2004. Antisnake venom activity of ethanolic seed extract of *Strychnos nux vomica* Linn. Indian Journal of Experimental Biology 42 (5), 468-475.

Chaves, F., Chacon, M., Badilla, B., Arevalo, C., 2007. Effect of *Ehinaceae purpurea* (Asteraceae) aqueous extract on antibody responses to *Bothrops asper* venom and immune cell response. Revista de Biologia Tropical 55(1), 113-119.

Chethankumar, M., Srinivas, L., 2008. New biological activity against phospholipase A2 by Turmerin, a protein from *Curcuma longa* L. *Biological Chemistry* 389(3), 299-303.

Chilpa, R.R., Garibay, F.R., Quijano, L., Guerrero, G.A.M., Ríos, T., 1994. Preliminary results on the protective effect of -(edunol), a pterocarpan from *Brongniartia podalyrioides* (Leguminosae), against *Bothrops atrox* venom in mice. Journal of Ethnopharmacology 42, 199-203.

Dalbelo,C.A., Colares, A.V., Leite, G.B., Ticli, F.K., Sampaio, S.V., Cintra, A.C., Rodrigues –Simioni, L., Dos Santosh. M.G., 2008. Antineurotoxic activity of *Galactia glauscences* against *Crotalus durissus* terrificus venom. Fitoterapia 79,378.

daSilva, J.O, Coppede, J.S., Frnandes, V.C., Santana, C.D., Ticli, F.K., Mazzi, M.V., Giglio, J.R., Periera, P.S., Soares, A.M., Sampaio, S.V., 2005. Anti-hemorrhagic, antinucleolytic and other antiophidian properties of the aqueous extract from *Pentaclethra macroloba.* Journal of Ethnopharmacology 100, 145-152.

daSilva, J.O., Fernandes, R.S., Ticli, F.K., Oliviera, C.Z., Mazzi, M.V., Franco, J.J., Giuliatti, S., Perreira, P.S., Soares, A.M., Sampaio, S.V., 2007. Triterpenoid saponins, new mettaloprotese snake venom inhibitors isolated from *Pentaclethra macroloba. Toxicon* 50, 283-291.

daSilva, S.L., Calgarrot, A.K., Chaar, J.S., Marangoni, S., 2008. Isolation and characterization of ellagic acid derivatives isolated from *Casearia sylvestris* S.W. aqueous extract with anti-PLA2 activity. Toxicon 52 (6),655-666.

Deepa, M., Gowda, T.V., 2006. Purification of a post-synaptic neurotoxic Phospholipase A2 from *Naja naja* venom and its inhibition by a glycoprotein from *Withania somnifera. Biochimie* 88 (6), 701-710.

Esmeraldino, L.E., Souza, A.M., Sampaio, S.V., 2005. Evaluation of aqueous extract of *Croton urucurana* Baillon (Euphorbiaceae) on the hemorrhagic activity induced by the venom of *Bothrops jararaca*, using new techniques to quantify hemorrhagic activity in rat skin*. Phytomedicine* 12 (8), 570-576.

Ferreira, L.A., Henriques, O.B., Andreoni, A.A., Vital, G.R., Campos, M.M., Habermehl, G.G., de, Moares, V.L., 1992. Antivenom and biological effects of ar-turmerone isolated from *Curcuma longa* (Zingiberaceae). *Toxicon* 30 (12), 1211-1218.

Francischetti, I.M., Monteiro, R.Q., Guimaraes, J.A., Francischetti, B., 1997. Identification of glycyrrhizin as a thrombin inhibitor. Biochemical and Biophysical Research Communications 235 (1), 259-263.

Girish, K.S., Kemparaju, K., 2005. Inhibition of *Naja naja* venom hyaluronidase by plant derived bioactive components and polysaccharides, Biochemistry. Biokhimia 70 (8), 948-952.

Girish, K.S., Mohanakumari, H.P., Nagaraju, S., Vishwanath, B.S., Kemparaju K., 2004. Hyalurinodase and protease activities from Indian snakes venoms; neutralization by *Mimosa pudica* root extract. *Fitoterapia* 75(3-4), 378-380.

Gomes, A., Das, R., Sarkhel, S., Mishra, R., Mukherjee, S., Bhattacharya, S., and Gomes A., 2010. Herbs and herbal constituents active against snakebites. *Indian Journal of Experimental Biology* 48, 865-878.

Gomes, A., Saha, A., Chatterjee, I., Chakravarty, A.K., 2007. Viper and Cobra venom neutralization by beta-sitosterol and stigmasterol isolated from the root extract of *Puchea indica* Less.(Asteraceae). *Phytomedicine* 14 (9), 637-643.

Guerranti, R., Aguiyi, J.C., Ogueli, I.G., Onorati, G., Neri, S., Rosati, F., DelBuono, F., Lampariello, R., Pagani, R., Marinello, E., 2004. Protection of *Mucuna pruriens* seeds against *Echis carinatus* venom is exerted through a multiform glycoprotein whose oligosaccharide chains are functional in this role. *Biochemical and Biophysical Research Communications* 323(2), 484-490.

Houghton, P.J., Osibogun, I.M., Bansal, S., 1992. A peptide from *Schumanniophyton magnificum* with anti-cobra venom activity. *Planta Medica* 58 (3), 263-265.

Houghton, P.J., Skari, K.P., 1994. The effect of blood clotting of some west Africa and plants used against snakebite. Journal of Ethnopharmacology 44 (2), 99-108.

Hung, Y.C., Sava, V., Hong, M.Y., Huang, G.S., 2004. Inhibitory effects on Phospholipase A2 and antivenin activity of melanin extracted from *Thea sinesis* Linn. *Life Sciences* 74 (16), 2037-2047.

Izidoro, L.F., Rodrigues, V.M., Rodrigues, R.S., Ferro, R.V., Hamaguchi, A., Giglio, J.R., Homsi-Brandeburgo, M.I., 2003. Neutralization of some hematological and hemostatic alterations induced by neuwiedase, a metalloproteinase isolated from *Bothrops neuwiedi* pauloensis snake venom by the extract from *Casearia mariquitensis* (Flacourtiaceae). *Biochimie* 85 (7), 669-675.

Januario, A.H., Santos, S.L., Marcussi, S., Mazz, M.V., Pietro, R.C., Sato, D.N., Ellena, J., Sampaio, S.V., Franca, S.C., Soares, A.M., 2004. Neo-clerodane diterpenoid, a new mettaloprotease snake venom inhibitor from *Baccharis trimera* (Asteraceae): anti-proteolytic and anti-hemorrhagic properties. *Chemico-Biological Interaction* 150 (3), 243-251.

John S., Premendran et al., 2011. Anti-cobra venom activity of Plant *Andrographis Peniculata* and its coparison with polyvalent anti-snake. Journal of Natural Sciences, Biology and Medicine 2 (2), 198-204.

Kini, R.M., Gowda, T., 1982. Studies on snake venoms Enzymes: Part 1. Purification of ATPase, a toxic component of *Naja naja* venom, and its inhibition by Potassium gymnemate. *Indian Journal of Biochemistry and Biophysics* 19 (2), 152-154.

Lobo, R., Punitha, ISR., Rajendran, K., Shirwaikar, A., 2006. Preliminary study on the anti-snake venom activity of alcoholic root extract of *Clerodendrum viscosum* (Vent). in *Naja naja* venom. Natural Product Science 12(3), 153-156.

Machiah, D.K., Girish, K.S., Gowda, T.V., 2006. A glycoprotein from a folk medicinal plant, *Withania somnifera* inhibits hyaluronidase activity of snake venoms. Comparative Biochemistry Physiology, Part-C. *Toxicol Pharmacology* 143(2), 158-161.

Mahanta, M., Mukherjee, A.K., 2001. Neutralization of lethality, myotoxicity, and toxic enzymes of *Naja kaouthia* venom by *Mimosa pudica* root extracts. *Journal of Ethnopharmacology* 75 (1), 55-60.

Mahanta, M., Mukherjee, A.K., 2001. Neutralization of lethality, myotoxicity and toxic enzymes of *Naja Kaouthia* venom by *Mimosa pudica* root extracts. Journal of Ethnopharmacology 75 (1), 55-60.

Makhija, I.K., Khamar, D., 2010. Anti-snake venom properties of medicinal plants. Scholars Research Library, Der *Pharmacia Lettre* 2 (5), 399-411.

Martz, W., 1992. Plants with a reputation against snakebite. Toxicon 30 (10), 1131-1142.

Meenatchisundaram, S., Parameshwari, G., Michael, A., 2009. Studies on anti-venom activity of *Andrographis penculata* and *Aristolochia indica* plant extract against *Daboia russeli* venom by *in vivo* and *in vitro* methods. S. Indian Journal of Technology 2 (4), 76-79.

Melo, P.A., Nascimento, M.C., Mors, W.B.,Suarez-Kurtz, G., 1994. Inhibition of the myotoxic and hemorrhagic activities of crotalid venoms by *Eclipta Prostrata* (Asteraceae) extracts and constituents. Toxicon 32(5), 595-603.

Memmi, A., Sansa, G., Rjeibi, I., El Ayeb, M., Srairi-Abid, N., Bellasfer, Z., Fekhih, A., 2007. Use of medicinal plants against scorpions and ophidian venoms. *Archives de Institut Pastuer de Tunis* 84 (1-4), 49-55.

Mendes, M.M., Oliviera, C.F., Lopes, D.S., Vale, L.H., Alcantara, T.M., Izidoro, L.F., et al., 2008. Anti-snake venom properties of *Schizolobium parahyba* (Caesalpinoidae) aqueous leaves extract. Phytotherapy Research 22 (7), 859-866.

Mors, W.B., do Nascimexito, M.C., Parente, J.P., da Silva, M.H., Melo, P.A., Suarez –Kurtz, G., 1989. Neutralization of lethal and myotoxic activities of South American rattlesnake venom by extracts and constituents of the plant *Eclipta prostrata* (Asteraceae). *Toxicon* 27(9), 1003-1009.

Mors, W.B., Nascimento, M.C., Perreira, B.M., Pereira, N.A., 2000. Plant natural products active against snakebite- the molecular approach. *Phytochemistry* 55 (6), 627-642.

Mostafa, M., Nahar, N., Mosihuzzaman, M., Sokeng, S. D., Fatima, N., Atta-Ur- Rahman., et al. (2006). Phosphodiesterase-I inhibitor quinovic acid glycosides from Bridelia ndellensis. Nat Prod Res. 20 (7), 686–92. doi:10.1080/14786410600661658

Mukherjee, A.K., Doley, R., Saikia, D., 2008. Isolation of a snake venom phospholipase A2 (PLA2) inhibitor (AIPLAI) from leaves of *Azadirachta indica* (Neem): Mechanism of PLA2 inhibition by AIPLAI in vitro condition. *Toxicon* 51 (8), 1548-1553.

Nakagawa, M., Nakanishi, K., Darko, L.L., Vick, J.A., 1982. Structures of cabenegrins A-1 and A-2, potent antisnake venoms. *Tetrahedron Letters* 23, 3855-3858.

Nataraju, A., Raghavendra Gowda, C.D., Rajesh, R., Vishwanath, B.S., 2007. Group 2 A Secretory PLA2 Inhibition by ursolic acid: a potent anti-inflammatory molecule. Current Topics of Medicinal Chemistry 7 (8), 801-809.

Nirmal, N., Praba, G.O., Velmurugan, D., 2008. Modeling studies on Phospholipase A2 –inhibitor complexes*. Indian Journal of Biochemistry and Biophysics* 45 (4), 256-262.

Nishijima, C.M., Rodrigues, C.M., Silva, A.M., Lopes-Ferreira, M., Vilegas, W., Hiruma-Lima, C.A., 2009. Anti-hemorrhagic activity of four Brazilian vegetables against Bothrops jararaca venom. Molecules 14 (3), 1072-1080.

Nunez, V., Castro, V., Murillo, R. , Ponce-Soto, L. A., Merfort e, I., Lomonte, B., (2005) Inhibitory effects of Piper umbellatum and Piper peltatum extracts towards myotoxic phospholipases A2 from Bothrops snake venoms: Isolation of 4-nerolidylcatechol as active principle, Phytochemistry 66, 1017–1025

Ode, O.J., Asuzu, U.I., 2006. The anti-snake venom activities of the methanolic extract of the bulb of *Crinum jagus* (Amarylladaceae). *Toxicon* 48 (3), 331-342.

Okonogi, T., Hattori, Z., Ogiso, A., and Mitsui, S. (1979). Detoxification by persimmon tannin of snake venoms and bacterial toxins. Toxicone 17 (5), 524–527. doi:10.1016/0041-0101(79)90287-3

Otero, R., Nunez, V., Barona, J., Fonnegra, R., Jimenez, S.L., Osorio, R.G., Salarriaga, M., Diaz, A., 2000. Snakebites and ethnobotany in the northwest region of Colombia; Part 3; Neutralization of hemorrhagic effect of Bothrops *atrox* venom. *Journal of Ethnopharmacology* 73 (1-2), 233-241.

Pereira, N.A., Ruppelt, B.M., Nascimento, M.C., Parente, J.P., Mors, W.B., 1991. An update on Plants used against snakebite. Brasilanisch-Deutsches Symposium fur Naturstoffchemie, Hanover, 48-51.

Pithayanukul, P., Laovachirasuwan, S., Bavovada, R., Pakmanee, N., Suttisri, R., 2004. Antivenom potential of butanolic extract of *Eclipta prostrata* against Malyalan pit viper. *Journal of Ethnopharmacology* 90 (2-3), 347-352.

Raslan, D.S., Jamal, C.M. Duarte, D.S., Borges, M.H., Lima De M.E.  (2002) Anti-PLA2 action test of Casearia sylvestris Sw Bollettino Chimico Farmaceutico, 141 (2002), pp. 457-460

Ruppelt, B.M., Pereira, E.F., Gon alves, L.C., Pereira, N.A., 1991. Pharmacological screening of plants recommended by folk medicine as snake-anti venom-1.Analgesic and anti-inflammatory activities. Memorias do Instito Oswaldo Cruz 86, 203-205.

Selvanayagam, Z.E., Gnanavendhan, S.G., Balakrishna, K., Rao, R.B., Sivaraman, J., Subramanian, K., Puri, R.K., 1996. Ehretianone, a novel quinonoid xanthene from *Ehretia buxifolia* with antisnake venom activity. *Journal of Natural Product* 59 (7), 664-667.

Shekhar, G. J., Penchala Pratap et al., 2011. Ethnic information on treatments for snakebites in Kadapa district of Andhra Pradesh, Life Sciences (12), 368-375.

Shirwaikar, A., Rajendran, K., Bodla, R., Kumar, C.D., 2004. Neutralization potential of *Viper russeli* (*Russels viper*) venom by ethanol leaf extract of *Acalypha indica L*. *Journal of Ethnopharmacology* 94 (2-3), 267-273.

Ticli, F.K., Hage, L.I., Cambraia, R.S., Pereira, P.S., Magro, A.J., Fontes, M.R., Stabeli, R.G., Franca, S.C., Giglio, J.R., Soares, A.M., Sampaio, S.V., 2005. Rosmarinic acid, a new snake venom phospholipase A2 inhibitor from *Cordia verbenaceae* (Boraginaceae): antiserum action potentiation and molecular interaction. *Toxicon* 46(3), 318-327.

Upasani, S.V., Beldar, V.G., Tatiya, A.U., Upasani, M.S., Surana, S.J., Patil, D.S., 2017. Ethnomedicinal Plants used for Snakebite in India:a brief overview. Integrative Medicine Research 6, 114-130.

Ushanandini, S., Nagaraju ,S., Harish, K..K..,Vedavathi, M., Machiah, D.K., Kemparaju, K.., Vishwanath, B.S., Gowda, T.V., Girish, K..S., 2006. The anti-snake venom properties of *Tamarindus indica* (leguminosae) seed extract. Phytotherapy Research 20 (10), 851-858.

Usubillaga, N., Khouri, S., Cedillo-Vaz , Yibirin, E., 2005. Anti snake venom effect of *Aristolochia odoratissima* L. aqueous extract on mice. *Acta Horticulture* 85 (3), 677. <http://dx.doi.org/10.17660/actahortic.2005.677.11>.

Vale, L.H., Mendes, M.M., Hamaguchi, A., Soares ,A.M., Rodrigues, V.M., Homsi-Br , eburgo, M.I., 2008. Neutralization of pharmacological and toxic activities of *Bothrops* snake venoms by *Schizolobium parahyba* (Fabaceae) aqeous extract and its fractions. Basic Clinical Pharmacology Toxicol 103 (1), 104-107.

Vishwanath, B.S., Appu, Rao, A.G., and Gowda, T.V. (1987). Interaction of phospholipase A from Vipera russelli with aristolochic acid: a circular dicroism study. Toxicon, 25: 939-946.

Wagner, H., Geyer, B,. Kiso, Y., Hikino, H., Rao, G.S., 1986. Coumestans as the main active principles of the liver drugs *Eclipta alba* and *Wedelia calendulaceae* 1. *Planta Medica* (5), 370-374.

Wagner, H., Prokch,A., 1985. Immunostimulatory drugs of fungi and higher plants, in *Economic and medicinal plant research,* edited by Wagner, H., Hikino, H., Fransworth, N.R., (Academic Press,London).113.

Yang, L.C., Wang, F., Liu, M., 1998. A study of an endothelin antagonist from a Chinese anti-snake venom medicinal herb. *Journal of Cardiovascular Pharmacology* 31 Suppl 1:S 249-250.

Adzu B, Abubakar MS, Izebe KS, Akumka DD, Gamaniel KS. 2004 Effect of Annona senegalensis rootbark extracts on *Naja nigricotlis* venom in rats. J Ethnopharmacol. 2005 Jan 15;96(3):507-13. doi: 10.1016/j.jep.2004.09.055. Epub 2004 Dec 8. PMID: 15619571.

Mahadeswaraswamy, Y. H., Sannaningaiah, D., Kumar, M., Goutham, Y., Kemparaju, K. 2009. Inhibition of local effects of Indian Daboia/Vipera russelli venom by the methanolic extract of grape (*Vitis vinifera* L.) seeds. Indian journal of biochemistry & biophysics. 46. 154-60. [Inhibition of local effects of Indian Daboia/Vipera russelli venom by the methanolic extract of grape (Vitis vinifera L.) seeds](https://www.researchgate.net/publication/26284119_Inhibition_of_local_effects_of_Indian_DaboiaVipera_russelli_venom_by_the_methanolic_extract_of_grape_Vitis_vinifera_L_seeds?_tp=eyJjb250ZXh0Ijp7ImZpcnN0UGFnZSI6InB1YmxpY2F0aW9uIiwicGFnZSI6InB1YmxpY2F0aW9uIn19)
